# Healthcare resource utilization and production loss in vedolizumab-treated inflammatory bowel disease patients: results from the Swedish prospective multicentre SVEAH study

**DOI:** 10.1177/17562848251352023

**Published:** 2025-07-17

**Authors:** Linda Ryen, Isabella Visuri, Sara Karlqvist, Carolina Malmgren, Daniel Bergemalm, Carl Eriksson

**Affiliations:** University Health Care Research Center, Faculty of Medicine and Health, Örebro University, Örebro, Sweden; Department of Gastroenterology, Faculty of Medicine and Health, Örebro University, Örebro, Sweden; Department of Gastroenterology, Faculty of Medicine and Health, Örebro University, Örebro, Sweden; Takeda Pharma AB, Stockholm, Sweden; Department of Gastroenterology, Faculty of Medicine and Health, Örebro University, Örebro, Sweden; Department of Gastroenterology, Faculty of Medicine and Health, Örebro University, Södra Grev Rosengatan, Örebro 70185, Sweden; Clinical Epidemiology Division, Department of Medicine Solna, Karolinska Institutet, Stockholm, Sweden

**Keywords:** Crohn’s disease, healthcare resource utilization, inflammatory bowel disease, ulcerative colitis, vedolizumab

## Abstract

**Background:**

Data on direct and indirect annual costs for inflammatory bowel disease (IBD) patients treated with vedolizumab are limited.

**Objectives:**

To evaluate the total annual direct healthcare costs and indirect costs among IBD patients treated with vedolizumab.

**Design:**

A prospective observational multicentre study involving 286 patients with Crohn’s disease (CD; *n* = 169) or ulcerative colitis (UC; *n* = 117) who started vedolizumab therapy during 2015–2017 at 21 hospitals across Sweden.

**Methods:**

Data on direct and indirect costs were collected during a 3-year follow-up period. Direct costs were measured as healthcare resource utilization including medication, hospital admissions and hospital-based outpatient visits. Indirect societal costs were measured as production losses from sick leave and disability pension. Data were obtained from the Swedish Quality Register for IBD and through linkage with national registers. Data are presented both for patients who continued treatment throughout the follow-up period and for patients who discontinued treatment (CD: *n* = 83; UC: *n* = 48).

**Results:**

The mean annual direct follow-up cost was €24,305 for all IBD patients, €24,873 for CD patients and €23,484 for UC patients (*p* = 0.24). No difference was observed between men and women (€24,506 vs €24,080; *p* = 0.87). Direct costs were similar in patients who continued vedolizumab for the entire study period (€24,401) and those who discontinued treatment (€24,192; *p* = 0.12). Medication was the primary driver of direct costs (64%), followed by hospital admissions (19%) and outpatient care (17%). Mean indirect costs were lower among patients who continued vedolizumab (€3044) than among those who stopped the treatment (€8927; *p* < 0.01). Increased direct costs were associated with perianal disease and high baseline disease activity in CD, and concurrent use of immunomodulators in UC.

**Conclusion:**

Patients treated with vedolizumab in Swedish clinical practice represent a group with high direct costs, primarily due to medication expenses. However, indirect costs were significantly lower than in previous reports.

## Plain language summary

Healthcare use and productivity loss in inflammatory bowel disease patients treated with vedolizumab: results from the Swedish SVEAH study

Inflammatory bowel disease (IBD) is a chronic idiopathic inflammatory disorder of the gastrointestinal tract. The two main types of IBD are Crohn’s disease and ulcerative colitis. IBD causes significant illness and can lead to complications like bowel blockages, fistulas, infections, and cancer, which create a heavy economic burden on society and healthcare. Vedolizumab is a biological treatment that was approved for treating IBD in 2014. A previous Swedish study, called the SVEAH study, showed that vedolizumab is effective in routine care. However, the cost of vedolizumab is higher than that of conventional therapies, costing over €10,000 per treatment year, depending on the country, healthcare system, and dosage.

The purpose of this study was to examine healthcare costs and productivity losses, such as sick leave, in patients treated with vedolizumab. To do this, we used the Swedish personal identification number to collect data from Swedish health registers for the participants in the SVEAH study.

We found that the average healthcare cost per patient was €24,305. Medication was the largest contributor to direct costs (64%), followed by hospital admissions (19%) and outpatient care (17%). Direct healthcare costs were similar for patients who continued vedolizumab throughout the study (€24,401) and those who stopped treatment (€24,192). However, productivity losses were lower for patients who continued vedolizumab (€3,044) than for those who stopped treatment (€8,927). High disease activity and the presence of perianal disease, such as abscesses or fistulas, were linked to higher costs in Crohn’s disease. Patients with ulcerative colitis who used immunomodulators alongside vedolizumab also had higher costs.

## Introduction

Inflammatory bowel disease (IBD) is a chronic idiopathic inflammatory disorder of the gastrointestinal tract. The two main types of IBD are Crohn’s disease (CD) and ulcerative colitis (UC). IBD is associated with significant morbidity and may lead to complications such as strictures, fistulas, infections and cancer, which impose a significant economic burden on society and the healthcare system. Although recent studies have shown a rapidly increasing incidence of IBD in newly industrialized countries in the Middle East, Asia and South America, the highest prevalence is still found in Northern Europe and North America.^1^ For example, in Örebro, Sweden, the prevalence of IBD in 2010 was 741 per 100,000 inhabitants.^2,3^

The primary aim of medical management is to induce and maintain remission, with the long-term goals of preventing disability, surgery and colorectal cancer.^4^ Biological agents have become the mainstay of therapy for patients with severe IBD and for those with mild to moderate disease who do not respond to or cannot tolerate conventional treatment.

In 2014, vedolizumab, a monoclonal antibody targeting α4β7 integrin, received approval from the European Medical Agency for treating moderate to severe CD and UC, based on evidence from the GEMINI studies.^5–7^ The prospective observational multicentre SVEAH study revealed significant clinical effectiveness of vedolizumab in Swedish routine care, even though 88% of the patients had been exposed to anti-tumour necrosis factor (anti-TNF) therapy at the start of treatment. However, vedolizumab is expensive, potentially exceeding €10,000 per treatment-year depending on the country, healthcare system and dosage.

Although biologics have proven effective in achieving clinical remission, enhancing work productivity and reducing hospitalization and surgery rates, there is evidence to suggest that healthcare costs related to IBD have increased over the past few decades, primarily due to escalating medication expenses.^8^ Conversely, it is well known that work disability is a common consequence of IBD. Previous studies have demonstrated that productivity losses represent a significant portion of the societal costs associated with IBD.^9^

In this study, we aimed to estimate the direct (medication, hospital admissions and costs for hospital-based outpatient visits) and indirect societal costs (productivity losses from sick leave and disability pension) for a well-characterized cohort of vedolizumab-treated patients with IBD.

## Materials and methods

### Patients

SVEAH was a nationwide prospective multicentre study conducted at 21 Swedish hospitals (13 regional and 8 university hospitals) between 2015 and 2021. The study population and design of the study are described in detail elsewhere.^10,11^ In short, eligible patients were ⩾18 years old and had active IBD at the start of vedolizumab. Active CD was defined as the presence of symptoms together with ulcers on colonoscopy or signs of active disease on magnetic resonance imaging, C-reactive protein (CRP) > lowest limit of detection, high-sensitivity CRP >2.87 mg/l or faecal calprotectin >200 µg/g. Active UC was defined as the presence of symptoms and a Mayo endoscopic subscore of ⩾2.^12^ Exclusion criteria were contraindications to vedolizumab or prior exposure to the drug.

Written informed consent was obtained from all participants included in the study. Vedolizumab was administrated intravenously. No predetermined dosing schedule was applied, and the treating physician was permitted to adjust the dose or dosing interval at any time.^10,11^ All patients were followed for 3 years. The reporting of this study conforms to the Strengthening the Reporting of Observational Studies in Epidemiology (STROBE) statement (Supplemental Material).^12^

### Direct costs

The patients were followed up using an electronic case report form (eCRF) integrated with the Swedish Quality Registry for Inflammatory Bowel Disease (SWIBREG).^13^ Through the use of the Swedish personal identity number,^14^ we were able to link the study population to other Swedish health data registers. Inpatient and outpatient data were retrieved from the National Patient Register, which has almost complete coverage of all non-primary care outpatient visits from 2001 onwards, including all outpatient visits to gastroenterologists, paediatric gastroenterologists and surgeons.^15,16^

Valuation of the resources used for providing inpatient and outpatient care was performed using the diagnosis-related groups (DRGs) constructed by the Swedish Association of Local Authorities and Regions. Each healthcare episode in the National Patient Register is categorized into a DRG code reflecting the amount of resources used considering diagnoses, treatments, procedures and length of hospital stay. Each DRG has a relative weight and an economic value assigned based on cost per patient administrative data.^17,18^ National retrospective weights from 2022 were used along with an economic value based on the 2022 agreement for debiting out-of-county patients in Region Örebro County. Costs for all healthcare were included in the calculations, not just costs related to IBD.

Data on medication use were collected through the study eCRF, as well as through linkage to SWIBREG^13^ and the Prescribed Drug Register, which ensured nearly 100% coverage.^19^ Non-prescription drugs were not included. Costs for prescription drugs were directly available from the Prescribed Drug Register, and the total cost was included in the estimations whether it was paid by the government or by the patient (prescription of medicines above SEK 2400 (approximately €200) per year is provided free of charge for the patient). For infusion drugs (vedolizumab, infliximab and the induction dose of ustekinumab), the coverage is insufficient, and data on their use were obtained from SWIBREG. Based on the number of doses from SWIBREG, the cost was estimated using the yearly prices negotiated at Örebro University Hospital.

All costs were converted to 2022 prices and presented in Euros. Cost estimations are presented in this article for years 1, 2 and 3 after inclusion in the study. To identify predictors of resource utilization, direct costs were analysed based on factors such as age, sex, disease duration, presence of extra-intestinal manifestations, Montreal classification and medical or surgical treatments. Additionally, the results were stratified based on baseline clinical disease activity (Harvey–Bradshaw index or partial Mayo Clinic score) and the reasons for initiating vedolizumab treatment, according to the following criteria: (1) patients with a primary non-response to the most recent anti-TNF therapy; (2) patients who discontinued the most recent anti-TNF due to adverse drug reactions, loss of response or other reasons and (3) patients naïve to anti-TNF.

### Indirect costs

Indirect costs were defined as production losses due to absence from paid work. Estimations were based on sick leave and disability pension payments provided by the Social Insurance Agency, which were available through the Swedish Longitudinal Integrated Database for Health Insurance and Labour Market Studies (LISA).^20^ The Swedish social insurance system offers compensation for both sick leave and disability pensions, either partially or fully. During the study period, no compensation was provided for the first day of sick leave, while days 2–14 were covered by the employer. Hence, only absences from work lasting longer than 14 days were included in the estimations.

The costs associated with productivity losses due to sick leave (>14 days) or permanent disability pension were estimated based on the total number of sick leave days, valued by the average monthly salary in Sweden for 2022, including social fees (€3575*1.3142), corresponding to a production loss of €154 per day of sick leave. The data in LISA are available for each calendar year. Since all costs were estimated annually based on each individual’s inclusion date, and thus do not correspond to calendar years, sick leave was assumed to be evenly distributed across the year. It should be noted that 1 day of sick leave does not necessarily correspond to 1 full working day; that is, a full-time worker and a part-time worker will both have 14 full days of sick leave if they are absent for 14 days. This introduces a risk of overestimating production losses; but on the other hand, absences shorter than 14 days were not included. Since there was a delay when producing LISA, information for 2020 was not available at the time of data retrieval for this study, and so sick leave data for patients entering the study in 2017 were not available for year 3 after inclusion. All patients of working age included in 2017 contributed data to the cost analyses for years 1 and 2 but were excluded from the analysis for year 3.

### Statistics

All costs are presented in Euro (€), inflation-adjusted to the year 2022. Continuous data are presented as mean with standard deviation (SD) or as median and interquartile range (IQR), and categorical data are expressed as frequencies and percentages. Differences between groups were assessed with the Mann–Whitney *U* test, Kruskal–Wallis test or chi-square test as appropriate. All tests were two-tailed, and *p*-values of <0.05 were considered statistically significant. Statistical analyses were performed using version 25.0 of IBM SPSS Statistics for Windows (IBM Corp., Armonk, NY, USA) or version 16.1 of STATA for Windows (StataCorp LLC, College Station, TX, USA).

## Results

**Figure 1.**
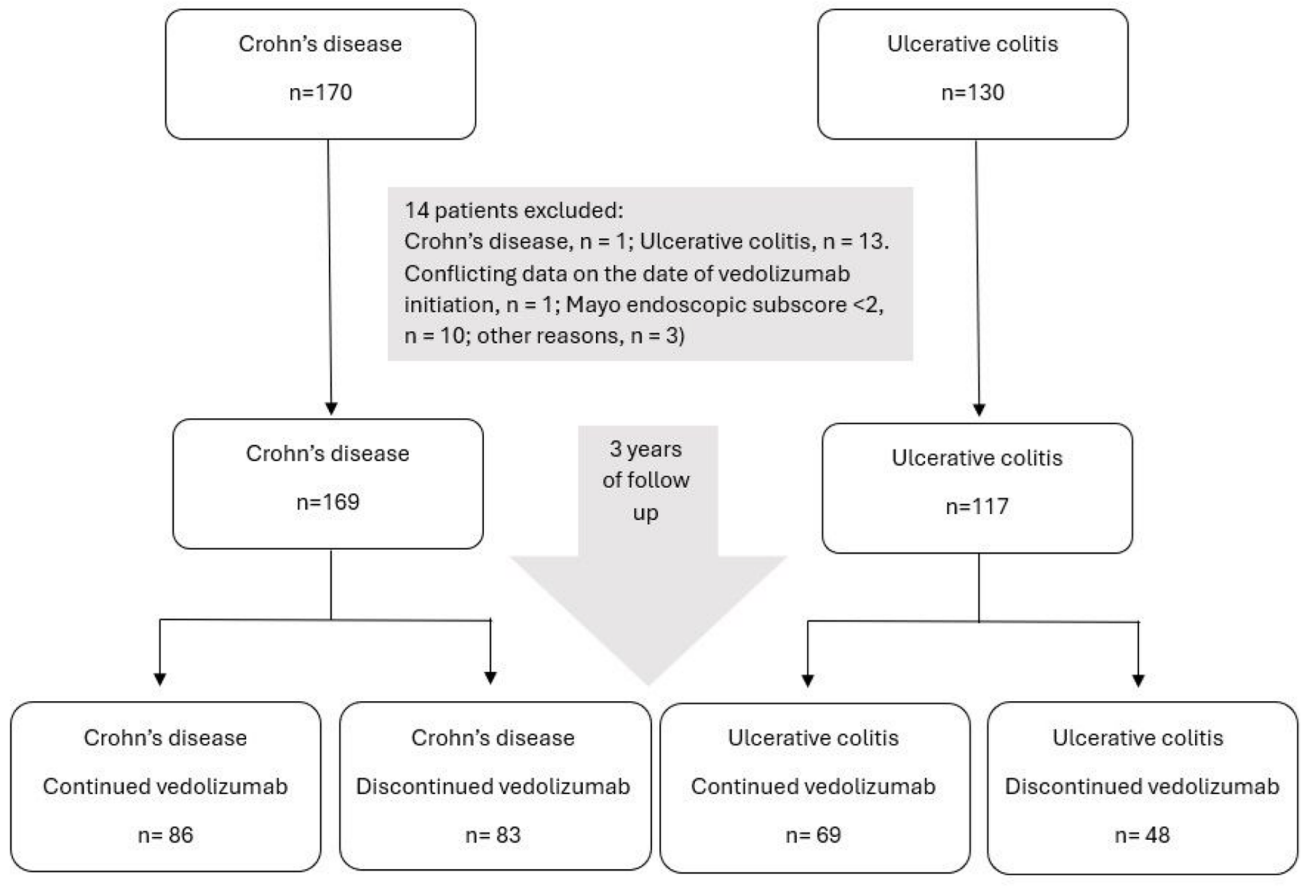
Flow diagram of the study.

The study population consisted of 286 patients with IBD (CD: *n* = 169; UC: *n* = 117) initiating vedolizumab between April 1, 2015 and November 31, 2017.^11,21^ Demographic and clinical characteristics of the study population are summarized in Table 1. During the 3-year follow-up period, 131 patients (CD: *n* = 83; UC: *n* = 48) discontinued the treatment for various reasons (evidence of its lack of or loss of response: *n* = 97; adverse drug reaction: *n* = 14; pregnancy: *n* = 4; other reasons: *n* = 16; Figure 1).

**Table 1.** Baseline demographics and clinical characteristics of patients with Crohn’s disease and ulcerative colitis.^21^

Variables	Crohn’s disease (*n* = 169)	Ulcerative colitis (*n* = 117)
Age in years, median (IQR)	43 (31–57)	41 (27–55)
Female sex	84 (50)	51 (44)
Disease duration in years, median (IQR)	9 (4–21)	5 (3–12)
Extra-intestinal manifestations	40 (24)	11 (9)
Current smoker	23 (14)	5 (4)
Location		
Ileal (L1)	28 (16)	
Colonic (L2)	52 (31)	
Ileocolonic (L3)	88 (52)	
Isolated upper GI (L4)	1 (1)	
Behaviour		
Inflammatory (B1)	88 (52)	
Stricturing (B2)	60 (36)	
Penetrating (B3)	21 (12)	
Perianal disease (p)	36 (21)	
Extent		
Proctitis (E1)		2 (2)
Left-sided colitis (E2)		28 (24)
Extensive colitis (E3)		87 (74)
Previous surgery	70 (41)	9 (8)^a^
Previous medications		
Immunomodulators	75 (44)	57 (49)
Anti-TNF therapy	150 (89)	101 (86)
Concurrent medication at baseline		
Aminosalicylates	11 (7)	43 (37)
Corticosteroids	44 (26)	28 (24)
Immunomodulators	20 (12)	28 (24)
Type of immunomodulator at baseline		
Azathioprine	12 (7)	22 (19)
Mercaptopurine	4 (2)	5 (4)
Methotrexate	4 (2)	1 (1)
Reasons for termination of last anti-TNF treatment^b^		
Primary non-response	30 (20)	38 (38)
Secondary loss of response	60 (40)	43 (43)
Adverse drug reaction	47 (31)	16 (16)
Other reasons	13 (9)	4 (4)
Prior anti-TNF substances		
⩾1	150 (89)	101 (86)
⩾2	84 (50)	42 (36)
⩾3	5 (3)	4 (3)

All data are given as *n* (%) unless otherwise indicated.

a Colectomy with ileorectal anastomosis.

b In total, 150 patients with Crohn’s disease and 101 patients with ulcerative colitis had previously stopped anti-TNF treatment.

Anti-TNF, anti-tumour necrosis factor; GI, gastrointestinal; IQR, interquartile range.

**Figure 2.**
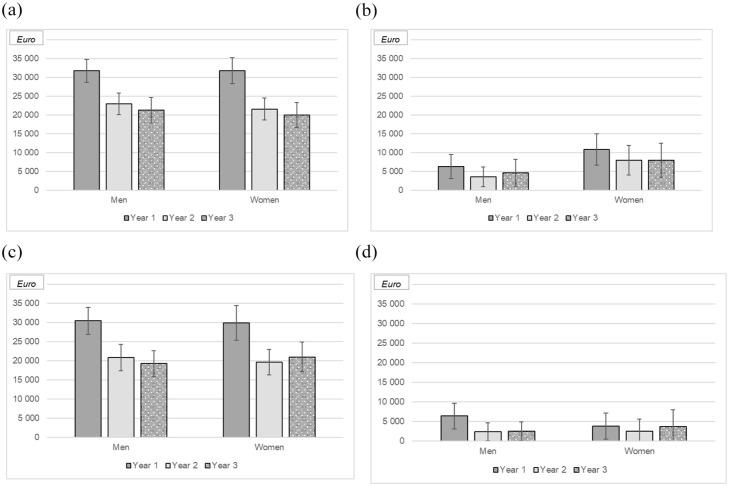
Mean total direct costs for men and women diagnosed with Crohn’s disease (CD) (a) or ulcerative colitis (UC) (c) and mean indirect costs (CD, b; UC, d), years 1–3 following start of treatment with vedolizumab. Error bars represent 95% confidence intervals.

The total direct healthcare cost for the cohort during the 3-year study period was €20,853,974, with €13,391,457 (64%) spent on medications (of which €12,802,692 (95%) was on biologics), €4,109,190 (20%) on hospital admissions and €3,353,327 (16%) on outpatient care (Figure 2(a)). The mean annual follow-up cost was €24,305 (SD: €10,391) for all individuals with IBD, €24,873 (SD: €10,917) for patients with CD and €23,484 (SD: €9566) for patients with UC (*p* = 0.24). No difference was observed between male (€24,506; SD: €10,331) and female patients (€24,080; SD: €10,491; *p* = 0.87) or between smokers (€22,269; SD: €10,073) and non-smokers (€24,432; SD: €10,434; *p* = 0.80; Figure 2).

Overall, the mean direct cost per patient-year of follow-up decreased from €31,126 (SD: €15,020) in year 1 to €21,454 (SD: €13,220) in year 2 (*p* < 0.01) and €20,226 (SD: €14,751) in year 3 (*p* = 0.04). Similar patterns were observed when stratifying by subtype of IBD (data not shown). The direct costs were similar in patients who continued vedolizumab for the entire study period (€24,401; SD: €8283) and those who discontinued treatment (€24,192; SD: €12,466; *p* = 0.12).

### Direct healthcare cost in patients with CD

**Figure 3.**
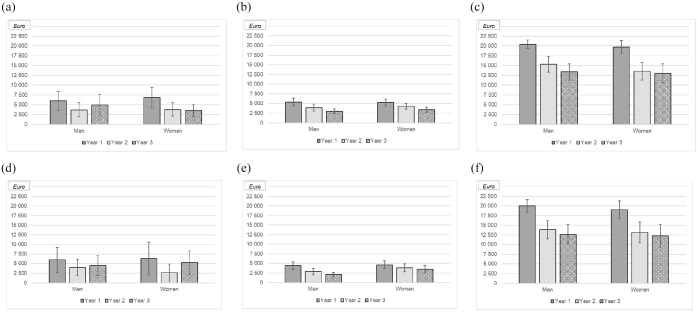
Mean cost of hospital admissions for patients with Crohn’s disease (CD) (a) and ulcerative colitis (UC) (d), outpatient visits (b, CD; e, UC) and medication (c, CD; f, UC) for men and women, years 1–3 following the start of treatment with vedolizumab. Error bars represent 95% confidence intervals.

During the 3-year follow-up, the total direct healthcare cost for the 169 patients with CD was €12,611,059, with €8,054,979 (64%) spent on medications (of which €7,856,458 was on biologics), €2,432,598 (19%) on hospital admissions and €2,123,482 (17%) on hospital-based outpatient care (Figure 3(a)–(c)). The median number of days admitted to hospital during the 3-year study period was 2 (IQR: 0–12), and the median number of visits to outpatient care (including doctor visits, nurse visits, infusion visits, dietician consultations and counsellor visits combined) was 18 (IQR: 10–30).

At the individual level, comparable annual follow-up costs were observed when the results were stratified by age, sex, smoking status, disease location, prior treatments and reasons for discontinuation of the last anti-TNF treatment (Table 2). In contrast, statistically significantly higher costs were observed among patients with perianal disease, in patients with moderate to severe clinical disease activity, and in patients without concurrent corticosteroids at initiation of therapy.

**Table 2.** Annual direct cost among patients with Crohn’s disease, divided by baseline demographics and clinical characteristics.

Variables	Crohn’s disease Annual cost, mean (SD)	*p*-Value^a^
Age		
⩽45 years	€24,946 (€10,940)	0.77
46–64 years	€25,011 (€10,099)	
⩾65 years	€24,302 (€12,791)	
Sex		
Male	€25,309 (€10,550)	0.35
Female	€24,433 (€11,323)	
Extra-intestinal manifestations		
Yes	€24,693 (€11,369)	0.75
No	€24,931 (€10,813)	
Current smoker		
Yes	€23,608 (€10,452)	0.63
No	€25,063 (€11,007)	
Location		
Ileal (L1)	€26,325 (€9636)	0.40
Colonic (L2)	€24,122 (€9771)	
Ileocolonic (L3)	€24,796 (€12,000)	
Behaviour		
Inflammatory (B1)	€24,236 (€9699)	0.20
Stricturing (B2)	€23,978 (€11,418)	
Penetrating (B3)	€30,095 (€13,280)	
Perianal disease		
Yes	€8,992 (€11,474)	<0.01
No	€23,759 (€10,530)	
Prior surgery		
Yes	€25,558 (€12,575)	0.79
No	€24,390 (€9612)	
Previous medications		
Immunomodulators		
Yes	€24,714 (€10,375)	0.62
No	€25,002 (€11,384)	
Anti-TNF therapy		
Yes	€24,506 (€10,194)	0.57
No	€27,775 (€15,558)	
Concurrent medication at baseline		
Aminosalicylates		
Yes	€23,113 (€9589)	0.36
No	€24,996 (€11,019)	
Corticosteroids		
Yes	€21,696 (€11,398)	0.05
No	€25,588 (€7829)	
Immunomodulators		
Yes	€27,315 (€10,558)	0.32
No	€24,546 (€10,957)	
Reasons for termination of the last anti-TNF treatment		
Primary non-response	€23,626 (€9025)	0.66
Secondary loss of response, adverse drug reaction, or other reason	€24,726 (€10,489)	
Bionaive	€27,775 (€15,558)	
Prior anti-TNF substances		
⩾1	€24,506 (€10,194)	0.73
⩾2	€24,074 (€10,101)	
⩾3	€31,873 (€17,803)	
Clinical disease activity at baseline		
Harvey–Bradshaw index ⩾8	€26,941 (€10,051)	<0.01
Harvey–Bradshaw index <8	€23,706 (€11,254)	

a Mann–Whitney *U*-test or Kruskal–Wallis test.

Anti-TNF, anti-tumour necrosis factor; SD, standard deviation.

### Direct healthcare cost in patients with ulcerative colitis

During the 3-year follow-up, the total direct healthcare cost for the 117 patients with UC was €8,242,916, with €5,336,478 (65%) spent on medications (of which €4,946,233 was on biologics), €1,676,592 (20%) on hospital admissions and €1,229,846 (15%) on hospital-based outpatient care (Figure 3(d) and (e)). The median number of days admitted to hospital during the 3-year study period was 1 (IQR: 0–10), and the median number of visits to outpatient care (nurse visits and doctor visits combined) was 14 (IQR: 8–26).

At the individual level, comparable annual follow-up costs were observed when the results were stratified by age, sex, smoking status, disease extent, prior treatments, clinical disease activity at baseline and reasons for discontinuation of the last anti-TNF treatment (Table 3). The only baseline variable that seemed to be associated with costs in patients with UC was the concurrent use of immunomodulator treatment.

**Table 3.** Annual direct cost among patients with ulcerative colitis, divided by baseline demographics and clinical characteristics.

Variables	Ulcerative colitis Annual cost, mean (SD)	*p*-Value^a^
Age		
⩽45 years	€22,924 (€9312)	0.38
46–64 years	€24,420 (€11,107)	
⩾65 years	€24,201 (€7493)	
Sex		
Male	€23,472 (€10,027)	0.31
Female	€23,500 (€9032)	
Extra-intestinal manifestations		
Yes	€20,722 (€6919)	0.50
No	€23,771 (€9780)	
Current smoker		
Yes	€20,761 (€8814)	0.87
No	€23,606 (€9617)	
Extent		
Proctitis (E1)	€24,473 (€945)	
Left-sided colitis (E2)	€25,633 (€10,808)	0.23
Extensive colitis (E3)	€22,770 (€9203)	
Prior surgery^b^		
Yes	€23,492 (€13,873)	0.99
No	€23,483 (€9209)	
Previous medications		
Immunomodulators		
Yes	€25,270 (€10,823)	0.17
No	€21,787 (€7919)	
Anti-TNF therapy		
Yes	€23,444 (€9802)	0.43
No	€23,738 (€8187)	
Concurrent medication at baseline		
Aminosalicylates		
Yes	€24,092 (€9340)	0.28
No	€23,131 (€9740)	
Corticosteroids		
Yes	€22,615 (€9459)	0.34
No	€23,757 (€9636)	
Immunomodulators		
Yes	€25,472 (€8453)	0.06
No	€22,859 (€9851)	
Reasons for termination of the last anti-TNF treatment		
Primary non-response	€24,155 (€9779)	0.63
Secondary loss of response, adverse drug reaction, or other reason	€23,015 (€9869)	
Bionaive	€23,738 (€8187)	
Prior anti-TNF substances		
⩾1	€23,444 (€8187)	0.65
⩾2	€24,383 (€11,961)	
⩾3	€28,958 (€14,717)	
Clinical disease activity at baseline		
Partial Mayo Clinic score ⩾5	€23,052 (€10,824)	0.35
Partial Mayo Clinic score <5	€23,938 (€8106)	

a Mann–Whitney *U*-test or Kruskal–Wallis test.

b Colectomy with ileorectal anastomosis.

Anti-TNF, anti-tumour necrosis factor; SD, standard deviation.

### Indirect societal costs

As the data in the LISA register have a long delay, we can only report indirect costs (productivity losses from sick leave and disability pension) for all patients during the first 2 years of follow-up. At study entry, 247 (86%) patients were of working age (<65 years). During the study period, the mean annual number of lost workdays was 46 (SD: 103; median: 0; IQR: 0–22) for patients with CD and 25 (SD: 69; median: 0; IQR: 0–9) for patients with UC, corresponding to mean productivity loss costs of €7098 (SD: €15,838) for patients with CD and €3775 (SD: €10,603) for patients with UC (*p* = 0.32; Figure 1). The number of sick days was unevenly distributed among the participants, and about 50% of participants had no sick days during the follow-up period.

No significant differences were observed between men (30 days; SD: 77; median: 0; IQR: 0–11 and €4612; SD: €11,826) and women (45 days; SD: 104; median: 0; IQR: 0–20 and €6999; SD: €16,061; *p* = 0.82).

In year 3, data on sick leave and disability pension were available for 180 (75%) of the 239 (84%) patients who were still of working age. Over the course of the study, the number of sick days declined from 53 (SD: 108; median: 0; IQR: 0–37) in year 1 to 33 (SD: 96; median: 0; IQR: 0–0) in year 2 and 37 (SD: 99; median: 0; IQR: 0–0) in year 3 (*p* < 0.01).

### Direct and indirect costs for patients with IBD who continued versus discontinued vedolizumab

The median treatment duration for the 131 patients who discontinued vedolizumab was 8.1 months (IQR: 3.8–15.3). The most common alternative treatments after discontinuation of vedolizumab included bowel surgery (*n* = 59), ustekinumab (*n* = 35), adalimumab (*n* = 20), golimumab (*n* = 15) infliximab (*n* = 8), tofacitinib (*n* = 7) and certolizumab pegol (*n* = 1) with each patient potentially receiving one or more of these treatments.

**Figure 4.**
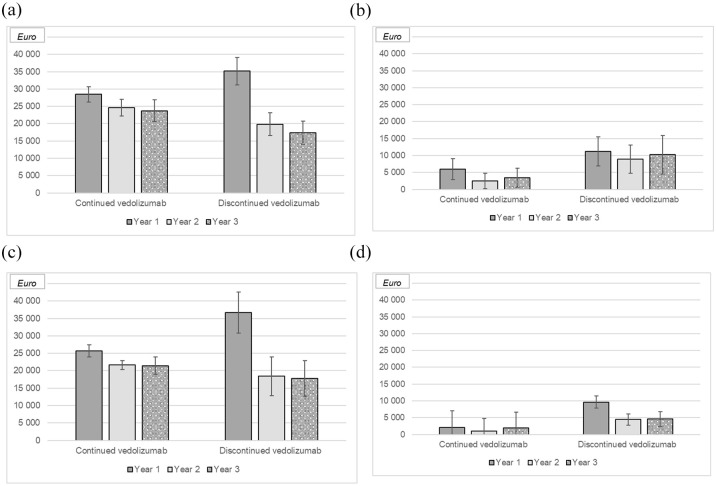
Mean total direct (a) and indirect (b) costs for patients with Crohn’s disease (CD) or ulcerative colitis (UC) (c and d) by continuation of vedolizumab, years 1–3 following start of treatment with vedolizumab. Error bars represent 95% confidence intervals.

No statistically significant difference in total direct healthcare costs was observed between patients who continued (€73,204; SD: €24,849) versus discontinued vedolizumab (€72,575; SD: €37,397; *p* = 0.122). However, there were substantial differences in how the costs were distributed: Patients who discontinued treatment had more outpatient visits, more days admitted to hospital, and underwent more major surgeries, whereas patients who continued treatment had higher medication costs (Table 4). Among patients continuing vedolizumab, the mean number of lost workdays and productivity loss costs was 20 days (SD: 64; median: 0; IQR: 0–1) and €3044 (SD: €9922), compared to 58 days (SD: 112; median: 0; IQR: 0–55) and €8927 (SD: € 17,171) among those who discontinued treatment (*p* < 0.01; Table 4; Figure 4). Overall, the total cost of production losses over the first 2 years was €3,282,528 for the entire cohort, comprising €943,583 for patients who continued vedolizumab (*n* = 155) and €2,338,945 for those who discontinued treatment (*n* = 131).

**Table 4.** Direct and indirect costs for patients with IBD who continued versus discontinued vedolizumab.

Variables	Continued vedolizumab (*n* = 155)		Discontinued vedolizumab (*n* = 131)		*p*-Value*
	Mean number (SD)	Mean cost (SD)	Mean number (SD)	Mean cost (SD)	
Hospital admissions, days	5 (12)		18 (38)		<0.01
Cost of hospital admissions^a^		€8142 (€27,021)		€21,734,(€25,932)	<0.01
Outpatient visits	18 (13)		25 (20)		<0.01
Costs of outpatient visits^b^		€10,157 (€7670)		€13,580 (€10,695)	0.04
Major surgery (e.g. small bowel resection or colectomy)^c^	0.12 (0.37)		0.67 (1.42)		<0.01
Cost of major surgeries		€1687 (€5486)		€7484 (€10,591)	<0.01
Minor surgery (e.g. surgery for perianal fistula)^c^	0.19 (0.61)		0.32 (1.63)		0.23
Cost of minor surgeries		€512 (€1784)		€578 (€3104)	0.20
Cost of biologics		€53,444 (€16,049)		€34,496 (€25,009)	<0.01
Total medication costs		€54,905 (€16,176)		€37,261 (€25,431)	<0.01
Total indirect costs		€3044 (€9922)		€8927 (€17,171)	<0.01
Number of lost workdays	20 (64)			58 (112)	<0.01

a The amount includes the costs of surgeries performed during the hospital stay.

b The cost includes expenses for day surgery procedures.

c The figures refer to the total number of surgeries during the entire study period.

* All *p*-values refer to cost comparisons and were calculated using the Mann–Whitney *U* test.

IBD, inflammatory bowel disease; No., number; SD, standard deviation.

## Discussion

In this prospective observational multicentre study, we assessed the total societal costs for a cohort of highly treatment-resistant IBD patients treated with vedolizumab, who represent a significant economic burden on the healthcare system. About 90% of this population had been exposed to anti-TNF therapy, and nearly half of those with CD had undergone at least one intestinal resection.

We found a high mean direct annual follow-up cost of €24,305, with the cost peaking in the first year of vedolizumab treatment and decreasing in the second and third years.^22^ Comparing patients who continued vedolizumab treatment (*n* = 155) with those who discontinued (*n* = 131), total direct healthcare costs did not differ significantly. However, substantial differences were observed in the cost distribution: patients who continued treatment had higher medication costs, whereas those who discontinued incurred higher costs related to hospital admissions, surgeries and outpatient visits. In contrast, patients who continued treatment had significantly lower indirect costs (i.e. costs due to productivity losses from sick leave and disability pensions) likely due to better disease control.

Unlike studies from the pre-biologic era,^23–25^ where surgery was the primary driver of healthcare costs, we found that medication constituted the largest portion (64%) of direct costs, while hospital admissions, including surgery costs, accounted for only 20% and outpatient care made up 16%. This aligns with other comprehensive reports.^9,26–28^ For example, the COIN study, which evaluated the cost of IBD across 14 Dutch hospitals, found that medication costs accounted for up to 71% of the cost in CD and 59% in UC, while hospitalization and surgery contributed 19% and less than 1%, respectively.^27^

IBD is associated with significant morbidity, and numerous studies have shown that costs related to sick leave and disability often surpass direct healthcare expenses.^9,29,30^

In a recent Swedish study, the total annual societal cost was estimated at $22,813 (≈ €21,444) for an average patient with CD, and $14,136 (≈ €13,288) for a patient with UC.^9^ These figures are somewhat lower than those in this study, which included a selected group of patients with therapy-refractory IBD. A key difference between the studies is that in the present study only about 15% of the total costs were indirect costs associated with sick leave and disability pension, compared to nearly 60% in the previous study.^9^ The SVEAH study demonstrated that vedolizumab had high clinical effectiveness in Swedish practice, with a steroid-free clinical remission rate of nearly 50% after 1 year.^11,21^ However, we cannot conclude from the current findings whether the drug itself reduced the need for sick leave and disability pension or whether the study population incidentally consisted of generally healthy individuals, apart from their IBD, with a high work capacity.

In CD, the presence of perianal disease and high disease activity were associated with slightly higher direct costs, while concurrent corticosteroid treatment at the start of vedolizumab surprisingly appeared to be linked to slightly lower costs. In patients with UC, costs were marginally higher for those using immunomodulators in addition to vedolizumab.

We had expected higher costs in individuals using corticosteroids, as steroid use is often a marker of more severe disease in observational studies (confounding by indication). However, there was a non-statistically significant association between corticosteroid use at treatment initiation and clinical remission after 52 weeks in the original SVEAH study (odds ratio: 2.04; 95% confidence interval: 0.84–4.91).^21^ We cannot provide a definitive explanation for this observation, but vedolizumab has a relatively long onset time in patients with CD, and corticosteroids may have prevented patients from prematurely discontinuing their treatment.

This study included a diverse group of patients from both regional hospitals and university centres, encompassing individuals who would have been excluded from the GEMINI trials, thereby reflecting the real-world use of vedolizumab in clinical practice. Other key strengths include the prospective data collection using an eCRF integrated within SWIBREG, and the availability of data from nationwide registers with comprehensive coverage.

By nature, cost-of-illness studies are descriptive and do not aim to offer direct information on the comparative cost-effectiveness of various treatments or to test specific hypotheses. However, by identifying and quantifying the relative contributions of different components to the overall economic burden of a disease, these studies can provide valuable insights into the potential cost implications of new treatments.

The main limitation of our study is the absence of data on items and services not captured in national registers, such as primary care visits, rehabilitation stay, sick leave periods shorter than 14 days and presenteeism (i.e. when employees are physically present at work but are unable to perform at full capacity due to illness). As a result, our cost estimates are likely to underestimate the true total costs. Unfortunately, the absence of a control group in this study limits the ability to compare the cost-effectiveness of different treatments based on our data. The relatively small sample size of our study population limited our ability to perform detailed cost analyses in smaller, yet clinically relevant subgroups, such as patients treated with combination therapies involving methotrexate or mercaptopurine, as well as those discontinuing vedolizumab due to adverse events. Additionally, since we used DRG codes instead of International Classification of Diseases (ICD) codes, we were unable to quantify the incidence of specific adverse events associated with vedolizumab. However, costs for managing these events (e.g. emergency care, hospitalizations, diagnostics and specialist visits) are included in the total cost estimates, as they are captured in the aggregated healthcare utilization through the DRG system. Using DRG weights to measure and value resource use may be perceived as imprecise because the estimated costs do not capture the full variation between patients. However, the DRG system in Sweden is clinically integrated and updated annually, with retrospective DRG weights designed to estimate resource use.^31^

## Conclusion

This is the first study to evaluate the mean annual cost for IBD patients treated with vedolizumab in Swedish clinical practice. High direct costs were seen, primarily driven by medication expenses, while indirect costs related to sick leave and disability pensions were lower than in previous studies, especially for patients continuing therapy. Increased costs were associated with perianal disease and high disease activity in CD, and with concomitant use of immunomodulators in UC.

## Supplemental Material

sj-docx-1-tag-10.1177_17562848251352023 – Supplemental material for Healthcare resource utilization and production loss in vedolizumab-treated inflammatory bowel disease patients: results from the Swedish prospective multicentre SVEAH study Supplemental material, sj-docx-1-tag-10.1177_17562848251352023 for Healthcare resource utilization and production loss in vedolizumab-treated inflammatory bowel disease patients: results from the Swedish prospective multicentre SVEAH study by Linda Ryen, Isabella Visuri, Sara Karlqvist, Carolina Malmgren, Daniel Bergemalm and Carl Eriksson in Therapeutic Advances in Gastroenterology

## Declarations

**Ethics approval and consent to participate:** The study was performed according to the ethical guidelines of the Declaration of Helsinki and was approved by the Regional Ethics Committee in Linköping, Sweden (2015/417-32). Written informed consent was obtained from all individual participants included in the study.

**Consent for publication:** Written informed consent was obtained from all individual participants included in the study.

**Competing interests:** L.R. has received grant support from Takeda. I.V. has served as a speaker for Takeda. S.K. has received lecture fees from Takeda. C.M. is an employee of Takeda. D.B. has served as a consultant for BMS, Janssen and Pharmacosmos and received speaker fees from BMS, Ferring, Janssen, Pharmacosmos, Pfizer, Sandoz and Takeda. C.E. received grant support/lecture fees/has served on the advisory board of BMS, Takeda, Janssen Cilag, Pfizer and AbbVie.

**Availability of data and materials:** The data underlying this article cannot be shared publicly due to legal restrictions, as they were obtained from a Swedish quality register. However, summary statistics can be provided on request.

## References

[1] Wang R Li Z Liu S , et al. Global, regional and national burden of inflammatory bowel disease in 204 countries and territories from 1990 to 2019: a systematic analysis based on the Global Burden of Disease Study 2019. BMJ Open 2023; 13(3): e065186. 10.1136/bmjopen-2022-065186 PMC10069527 36977543

[2] Eriksson C Cao Y Rundquist S , et al. Changes in medical management and colectomy rates: a population-based cohort study on the epidemiology and natural history of ulcerative colitis in Orebro, Sweden, 1963–2010. Aliment Pharmacol Ther 2017; 46(8):748–757. 28833287 10.1111/apt.14268

[3] Zhulina Y Udumyan R Henriksson I , et al. Temporal trends in non-stricturing and non-penetrating behaviour at diagnosis of Crohn’s disease in Orebro, Sweden: a population-based retrospective study. J Crohns Colitis 2014; 8(12): 1653–1660. 25113899 10.1016/j.crohns.2014.07.006

[4] Turner D Ricciuto A Lewis A , et al. STRIDE-II: an update on the Selecting Therapeutic Targets in Inflammatory Bowel Disease (STRIDE) Initiative of the International Organization for the Study of IBD (IOIBD): determining therapeutic goals for treat-to-target strategies in IBD. Gastroenterology 2021; 160(5): 1570–1583. 33359090 10.1053/j.gastro.2020.12.031

[5] Feagan BG Rutgeerts P Sands BE , et al. Vedolizumab as induction and maintenance therapy for ulcerative colitis. N Engl J Med 2013; 369(8): 699–710. 23964932 10.1056/NEJMoa1215734

[6] Sandborn WJ Feagan BG Rutgeerts P , et al. Vedolizumab as induction and maintenance therapy for Crohn’s disease. N Engl J Med 2013; 369(8): 711–721. 23964933 10.1056/NEJMoa1215739

[7] Sands BE Feagan BG Rutgeerts P , et al. Effects of vedolizumab induction therapy for patients with Crohn’s disease in whom tumor necrosis factor antagonist treatment failed. Gastroenterology 2014; 147(3): 618–627.e3. 10.1053/j.gastro.2014.05.008 24859203

[8] van Linschoten RCA Visser E Niehot CD , et al. Systematic review: societal cost of illness of inflammatory bowel disease is increasing due to biologics and varies between continents. Aliment Pharmacol Ther 2021; 54(3): 234–248. 34114667 10.1111/apt.16445 PMC8361769

[9] Khalili H Everhov ÅH Halfvarson J , et al. Healthcare use, work loss and total costs in incident and prevalent Crohn’s disease and ulcerative colitis: results from a nationwide study in Sweden. Aliment Pharmacol Ther 2020; 52(4): 655–668. 32902894 10.1111/apt.15889 PMC7490827

[10] Eriksson C Sun J Bryder M , et al. Impact of inflammatory bowel disease on the risk of acute coronary syndrome: a Swedish Nationwide Cohort Study. Aliment Pharmacol Ther 2024; 59: 1122–1133. 38425022 10.1111/apt.17932

[11] Visuri I Eriksson C Karlqvist S , et al. Long-term outcomes of vedolizumab in inflammatory bowel disease: the Swedish prospective multicentre SVEAH extension study. Therap Adv Gastroenterol 2023; 16: 17562848231174953. 10.1177/17562848231174953 PMC10236258 37274297

[12] von Elm E Altman DG Egger M , et al. Strengthening the reporting of observational studies in epidemiology (STROBE) statement: guidelines for reporting observational studies. BMJ 2007; 335(7624): 806–808. 17947786 10.1136/bmj.39335.541782.AD PMC2034723

[13] Ludvigsson JF Andersson M Bengtsson J , et al. Swedish Inflammatory Bowel Disease Register (SWIBREG) – a nationwide quality register. Scand J Gastroenterol 2019; 54(9): 1089–1101. 31498717 10.1080/00365521.2019.1660799

[14] Ludvigsson JF Otterblad-Olausson P Pettersson BU , et al. The Swedish personal identity number: possibilities and pitfalls in healthcare and medical research. Eur J Epidemiol 2009; 24(11): 659–667. 19504049 10.1007/s10654-009-9350-y PMC2773709

[15] Swedish National Board of Health and Welfare, et al. Kvalitet och innehåll i patientregistret. Utskrivningar från slutenvården 1964-2007 och besök i specialiserad öppenvård (exklusive primärvårdsbesök) 1997-2007.(Quality and content of the Patient Register) (2009-125-15), https://www.socialstyrelsen.se/contentassets/12ab370b4cec441db1b8b0c78b9d6a1d/2009-125-15_200912515_rev2.pdf (2009, accessed January 20, 2025).

[16] Ludvigsson JF Andersson E Ekbom A , et al. External review and validation of the Swedish national inpatient register. BMC Public Health 2011; 11: 450. 21658213 10.1186/1471-2458-11-450 PMC3142234

[17] Swedish Association of Local Authorities and Regions. National cost per patient principles, https://skr.se/download/18.187235b2180b4dcf16ac5ae/1652426603256/SKR_A4_KPP-principer_Webb.pdf (2020, accessed January 20, 2025).

[18] Swedish National Board of Health and Welfare. DRG – basic concept and principles, https://www.socialstyrelsen.se/globalassets/sharepoint-dokument/dokument-webb/klassifikationer-och-koder/drg-grundlaggande-begrepp-och-principer.pdf (2022, accessed 20 January 2025).

[19] Wettermark B Hammar N Fored CM , et al. The new Swedish Prescribed Drug Register – opportunities for pharmacoepidemiological research and experience from the first six months. Pharmacoepidemiol Drug Saf 2007; 16(7): 726–735. 16897791 10.1002/pds.1294

[20] Ludvigsson JF Svedberg P Olén O , et al. The longitudinal integrated database for health insurance and labour market studies (LISA) and its use in medical research. Eur J Epidemiol 2019; 34(4): 423–437. 30929112 10.1007/s10654-019-00511-8 PMC6451717

[21] Eriksson C Rundquist S Lykiardopoulos V , et al. Real-world effectiveness of vedolizumab in inflammatory bowel disease: week 52 results from the Swedish prospective multicentre SVEAH study. Therap Adv Gastroenterol 2021; 14: 17562848211023386. 10.1177/17562848211023386 PMC8255566 34276808

[22] Everhov ÅH Söderling J Befrits G , et al. Increasing healthcare costs in inflammatory bowel disease 2007–2020 in Sweden. Aliment Pharmacol Ther 2023; 58(7): 692–703. 37594381 10.1111/apt.17675

[23] Blomqvist P Ekbom A . Inflammatory bowel diseases: health care and costs in Sweden in 1994. Scand J Gastroenterol 1997; 32(11): 1134–1139. 9399395 10.3109/00365529709002993

[24] Bassi A Dodd S Williamson P , et al. Cost of illness of inflammatory bowel disease in the UK: a single centre retrospective study. Gut 2004; 53(10): 1471–1478. 15361497 10.1136/gut.2004.041616 PMC1774248

[25] Odes S Vardi H Friger M , et al. Cost analysis and cost determinants in a European inflammatory bowel disease inception cohort with 10 years of follow-up evaluation. Gastroenterology 2006; 131(3): 719–728. 16952541 10.1053/j.gastro.2006.05.052

[26] Lawton J Achit H Pouillon L , et al. Cost-of-illness of inflammatory bowel disease patients treated with anti-tumour necrosis factor: a French large single-centre experience. United European Gastroenterol J 2019; 7(7): 908–913. 10.1177/2050640619853448 PMC6683635 31428415

[27] van der Valk ME Mangen MJ Leenders M , et al. Healthcare costs of inflammatory bowel disease have shifted from hospitalisation and surgery towards anti-TNFα therapy: results from the COIN study. Gut 2014; 63(1): 72–79. 23135759 10.1136/gutjnl-2012-303376

[28] van der Valk ME Mangen MJ Severs M , et al. Evolution of costs of inflammatory bowel disease over two years of follow-up. PLoS One 2016; 11(4): e0142481. 10.1371/journal.pone.0142481 PMC4839678 27099937

[29] Holko P Kawalec P Mossakowska M , et al. Health-related quality of life impairment and indirect cost of Crohn’s disease: a self-report study in Poland. PLoS One 2016; 11(12): e0168586. 10.1371/journal.pone.0168586 PMC5161376 27992531

[30] Mesterton J Jönsson L Almer SH , et al. Resource use and societal costs for Crohn’s disease in Sweden. Inflamm Bowel Dis 2009; 15(12): 1882–1890. 19408336 10.1002/ibd.20939

[31] Špacírová Z Epstein D Espín J . Are costs derived from diagnosis-related groups suitable for use in economic evaluations? A comparison across nine European countries in the European Healthcare and Social Cost Database. Eur J Health Econ 2022; 23(9): 1563–1575. 35217963 10.1007/s10198-022-01444-y

